# Accuracy of Bolus and Basal Rate Delivery of Different Insulin Pump Systems

**DOI:** 10.1089/dia.2018.0376

**Published:** 2019-03-30

**Authors:** Guido Freckmann, Ulrike Kamecke, Delia Waldenmaier, Cornelia Haug, Ralph Ziegler

**Affiliations:** ^1^ Institut für Diabetes-Technologie, Forschungs- und Entwicklungsgesellschaft mbH an der Universität Ulm, Ulm, Germany.; ^2^ Diabetes Clinic for Children and Adolescents, Muenster, Germany.

**Keywords:** Continuous subcutaneous insulin infusion, Insulin pump, Accuracy, Basal rate, Bolus

## Abstract

***Background:*** Insulin pumps are used for basal rate and bolus insulin delivery in patients with diabetes. In this in vitro study, accuracy of delivery of different commercial insulin pumps was evaluated.

***Materials and Methods:*** Accuracy of 10 different insulin pump systems (5 durable pumps with different insulin infusion sets and 1 patch pump) was tested with a microgravimetric method. Mean bolus accuracy of 25 successive 1 U boluses and of 12 successive 10 U boluses was assessed, and delivery time for 10 U boluses was measured. Basal rate accuracy at 1.0 U/h was evaluated for 72 h and for individual 1-h windows.

***Results:*** Mean bolus delivery was within ±5% of target for both tested bolus sizes, but precision of individual boluses was higher with the larger boluses. Delivery times varied between the different pump models but agreed with the specifications of the respective manufacturers. Regarding basal rate accuracy, the total deviation for 72 h was very small in all pumps; however, larger deviations were observed during the first 12 h. For the patch pump, large variations between individual 1-h windows were observed.

***Conclusions:*** In general, all compared insulin pump systems showed a similar level of accuracy. Differences, especially between durable pumps and the patch pump, were observed when considering each hour of basal rate delivery separately.

## Introduction

Continuous subcutaneous insulin infusion (CSII) therapy is indicated for people with type 1 diabetes who fail to achieve an adequate glycemic control with multiple daily injections (MDI) and who are able and motivated to intensively manage their therapy, and for selected people with insulin-treated type 2 diabetes.^1^ Especially when combined with continuous glucose monitoring, it is an efficient and the most physiological therapy option with many benefits.^2^ Common insulin pumps allow a more flexible therapy than MDI, including a variable basal rate and different bolus types, and in one study they have been shown to deliver insulin more accurately than syringes or insulin pens.^3^ Yet, differences in delivery accuracy between available pumps have been reported, especially when comparing durable pumps to patch pumps.^4–7^ Regarding accuracy, one has to distinguish between bolus accuracy, when a rather large volume is delivered within a short time, and the quasi-continuously delivered basal rate accomplished by repeated small short bursts. In addition, the accuracy of an insulin pump depends among others on the actually used bolus or basal rate size and probably also on the used insulin infusion set (IIS). Furthermore, experimentally assessed accuracy might be influenced by an adequate test setting. The latter has previously been intensively discussed and was, therefore, addressed separately by us.^9^ In this study, accuracy of bolus and basal rate delivery was tested in vitro based on IEC 60601-2-24^8^ for different insulin pump systems available in Germany at the time of testing.

## Materials and Methods

### Insulin pump systems

Ten different insulin pump systems, that is, combinations of insulin pumps and IIS, were tested (Table 1). Five durable pumps with common IIS and one patch pump were included. All insulin pumps were prepared according to the respective manufacturers' instructions for use and filled with insulin aspart (NovoRapid^®^; Novo Nordisk A/S, Bagsværd, Denmark). From each insulin pump system three individual insulin pumps were tested in three repetitions to obtain nine data sets (runs) per system.

**Table 1.** Tested Insulin Pump Systems

*Pump system*	*Insulin pump*	*Manufacturer*	*Infusion set*	*Cannula*	*Tubing (cm)*
AI-F	Accu-Chek^®^ Insight	Roche Diabetes Care GmbH, Mannheim, Germany	Accu-Chek^®^ Insight Flex	6 mm Teflon	40
AI-R	Accu-Chek Insight		Accu-Chek^®^ Insight Rapid	6 mm Steel	40
ASC-F	Accu-Chek^®^ Spirit Combo	Roche Diabetes Care GmbH, Mannheim, Germany	Accu-Chek^®^ FlexLink	8 mm Teflon	60
ASC-R	Accu-Chek Spirit Combo		Accu-Chek^®^ Rapid-D Link	6 mm Steel	50
AV-I	Animas^®^ Vibe^®^	Animas Corporation, Inc., West Chester, PA, USA	inset™ II	6 mm Teflon	60
M6-Q	MiniMed^®^ 640G	Medtronic MiniMed, Northridge, CA, USA	MiniMed^®^ Quick-set^®a^	6 mm Teflon	46
MO	mylife™ OmniPod^®^	Insulet Corporation, Billerica, MA, USA	—	6.5 mm Teflon	—
PV-M	Paradigm^®^ VEO™	Medtronic MiniMed, Northridge, CA, USA	MiniMed^®^ Mio™	6 mm Teflon	46
PV-Q	Paradigm VEO		MiniMed Quick-set	6 mm Teflon	46
PV-S	Paradigm VEO		MiniMed^®^ Sure-T^®^	6 mm Steel	46

^a^ The batch used in this study was recalled by the manufacturer (Medtronic reference FA785) after the investigation. The results did not indicate any malfunction of the infusion sets used in this investigation; all product names and trademarks are the property of their respective owners.

### Test procedures

Test setups and procedures were described in detail before.^9^ In short, accuracy was determined by means of weight differences through insulin delivery into a water-filled oil-covered beaker placed on a balance as described in IEC 60601-2-24.^8^ For the patch pump, the setup had to be modified due to the lack of an administration set.^9^

Bolus accuracy was determined for a standard bolus of 1 and 10 U. For the 1 U bolus accuracy, 25 boluses were delivered and weighed individually. For the evaluation of the 10 U bolus, only 12 boluses were delivered due to the limited reservoir size of some pump systems that would have required refilling, which might have influenced the results. In addition, delivery time for a 10 U bolus was measured at standard speed settings with all systems. If a pump offered different bolus speed settings, all available speeds were tested, but only with one IIS per insulin pump.

Basal rate accuracy was determined for a constant basal rate of 1.0 U/h. The basal rate was run for 72 h and weight increases measured every 15 min were used for calculations of the basal rate accuracy.

### Data analysis

Bolus and basal rate delivery of U-100 insulin (100 U/mL) were calculated from weight increases, using a density of 1.005 g/mL for insulin aspart.^10^

For each individual bolus, deviation from target (1 or 10 U, respectively) was calculated. In addition, the percentage of boluses within different ranges from target (±15%, ±10%, ±5%) was calculated. The 72 h of basal rate delivery was divided into 1-h windows and deviation from target (1.0 U/h) was calculated for each 1-h window, and the percentage of 1-h windows within different ranges from target (±15%, ±10%, ±5%) was calculated.^9^ In addition, the total deviation for the whole 72 h and for the first 12 h was determined, respectively. Trumpet curves as required by IEC 60601-2-24 were generated by calculating the minimal and maximal deviation for observation windows of 15, 60, 150, 330, 570, and 930 min, respectively, whereas the first 24 h were neglected.

Data from the nine data sets per insulin pump system were evaluated separately but summarized for data presentation. Data were analyzed descriptively; no comparative analyses between the tested insulin pump systems were performed.

## Results

### Bolus accuracy and delivery time

**FIG. 1.**
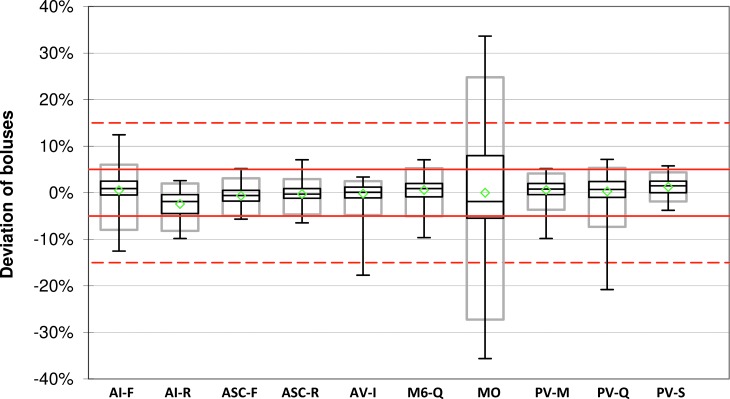
Bolus accuracy for 1 U boluses. For each system, mean (green diamonds), median with first and third quartile (black boxes), minimum and maximum (antennae), and 95% range (gray boxes) are shown (*n* = 225 boluses). Red lines and red dashed lines indicate target ±5% and target ±15%, respectively.

The mean deviation of the weighed 1 U boluses ranged from −2.4% ± 2.8% to +1.2% ± 1.7% of the target delivery among all insulin pump systems and 77%–100% of all delivered boluses per system were within ±15% of the intended bolus volume (Table 2 and Fig. 1). With all systems except for the patch pump MO, at least 95% of all 1 U boluses were within ±15% of target. With the mean values being similar between all tested pump systems, precision between the single boluses was slightly higher in PV-S (95% range: 0.06 U) and AV-I (95% range: 0.07 U) than in the other pumps (95% range from 0.08 U to 0.52 U) (Fig. 1).

**Table 2.** Bolus Accuracy: Mean Deviation from Target and Percentage of Individual Boluses Within ±15% of Target for 1 U (*n* = 225 Boluses) and 10 U Boluses (*n* = 108 Boluses)

*Pump system*	*1 U*		*10 U*	
	*Mean deviation ± SD*	*Individual boluses within ±15% (%)*	*Mean deviation ± SD*	*Individual boluses within ±15% (%)*
AI-F	+0.5% ± 3.5%	100.0	+0.3% ± 1.1%	100.0
AI-R	−2.4% ± 2.8%	100.0	+0.4% ± 0.9%	100.0
ASC-F	−0.6% ± 1.9%	100.0	−0.6% ± 0.9%	100.0
ASC-R	−0.3% ± 1.9%	100.0	−0.6% ± 0.8%	100.0
AV-I	−0.2% ± 2.1%	99.6	−0.9% ± 0.6%	100.0
M6-Q	+0.6% ± 2.6%	100.0	−0.7% ± 0.6%	100.0
MO	+0.0% ± 12.5%	76.9	+0.3% ± 0.7%	100.0
PV-M	+0.6% ± 2.1%	100.0	−0.8% ± 0.9%	100.0
PV-Q	+0.3% ± 3.3%	99.6	−0.5% ± 0.9%	100.0
PV-S	+1.2% ± 1.7%	100.0	−0.5% ± 0.9%	100.0

**FIG. 2.**
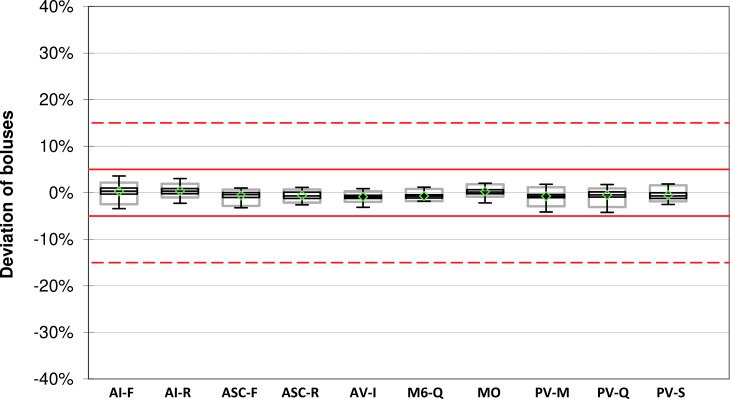
Bolus accuracy for 10 U boluses. For each system, mean (green diamonds), median with first and third quartile (black boxes), minimum and maximum (antennae), and 95% range (gray boxes) are shown (*n* = 108 boluses). Red lines and red dashed lines indicate target ±5% and target ±15%, respectively.

Upon delivery of 10 U boluses, all measured boluses of all systems were within ±5% of the intended bolus volume (Table 2 and Fig. 2).

Delivery times of a 10 U bolus ranged from 00:20 min:s to 06:39 min:s in the different systems at standard bolus speed settings and were reproducible within each system (Table 3). Standard speed was fastest for AV-I and slowest for M6-Q and MO; however, M6-Q also offered a faster option.

**Table 3.** Bolus Delivery Time for 10 U Boluses with Different Bolus Delivery Speeds (Mean of *n* = 9 Boluses)

	*Standard (min:s)*	*Very slow (min:s)*	*Slow (min:s)*	*Moderate (min:s)*	*Fast (min:s)*
AI-F	00:50	3:20	01:40	01:07	—
AI-R	00:50	—^a^	—^a^	—^a^	—
ASC-F	00:50	—	—	—	—
ASC-R	00:50	—	—	—	—
AV-I	00:20	—	00:52	—	—
M6-Q	06:38	—	—	—	00:40
MO	06:39	—	—	—	—
PV-M	04:58	—	—	—	—
PV-Q	04:58	—	—	—	—
PV-S	04:58	—	—	—	—

^a^ Not assessed with this insulin infusion set.

### Basal rate accuracy

The total deviation of the measured weight increase at the set basal rate of 1.0 U/h after 72 h was very small (between **−**0.1% and 1.4%) for all tested insulin pump systems (Table 4). During the first 12 h, larger deviations of up to 10% were observed. Most pumps showed an increased delivery at the beginning of the measurement that gradually decreased during the first 6–12 h. Although during the first 12 h, small differences between the individual systems could be seen with deviations ranging from 0.1% to 9.9%, differences were marginal afterward. For those insulin pumps that were tested with different IIS, no influence of the used IIS on delivery accuracy was observed.

**Table 4.** Basal Rate Accuracy (1.0 U/h): Total Deviation for Different Time Periods, Mean and Minimum and Maximum of Nine Repetitions

*Pump system*	*Total deviation (0–72 h)*	*Total deviation (0–12 h)*	*Total deviation (12–72 h)*
AI-F	0.0% (−0.52% to 0.70%)	0.6% (−2.26% to 5.10%)	−0.1% (−0.98% to 0.80%)
AI-R	1.0% (−0.48% to 2.38%)	0.1% (−4.10% to 4.63%)	1.1% (−0.21% to 2.04%)
ASC-F	−0.4% (−1.26% to 0.31%)	3.7% (−1.87% to 10.31%)	−1.3% (−1.73% to −0.57%)
ASC-R	0.2% (−0.81% to 0.73%)	3.0% (1.36% to 4.38%)	−0.3% (−1.25% to 0.12%)
AV-I	0.0% (−0.58% to 0.47%)	4.7% (1.72% to 6.71%)	−0.9% (−1.66% to −0.31%)
M6-Q	0.9% (0.11% to 2.02%)	9.9% (6.48% to 13.04%)	−0.9% (−1.65% to −0.19%)
MO	1.4% (0.15% to 2.60%)	5.0% (1.71% to 9.14%)	0.6% (−0.31% to 1.58%)
PV-M	−0.1% (−0.79% to 0.77%)	1.7% (−1.28% to 3.30%)	−0.5% (−1.12% to 0.26%)
PV-Q	0.1% (−0.86% to 0.67%)	2.9% (−2.83% to 6.51%)	−0.5% (−0.96% to −0.12%)
PV-S	0.5% (−0.29% to 1.45%)	2.9% (0.52% to 6.33%)	0.1% (−0.47% to 0.59%)

**FIG. 3.**
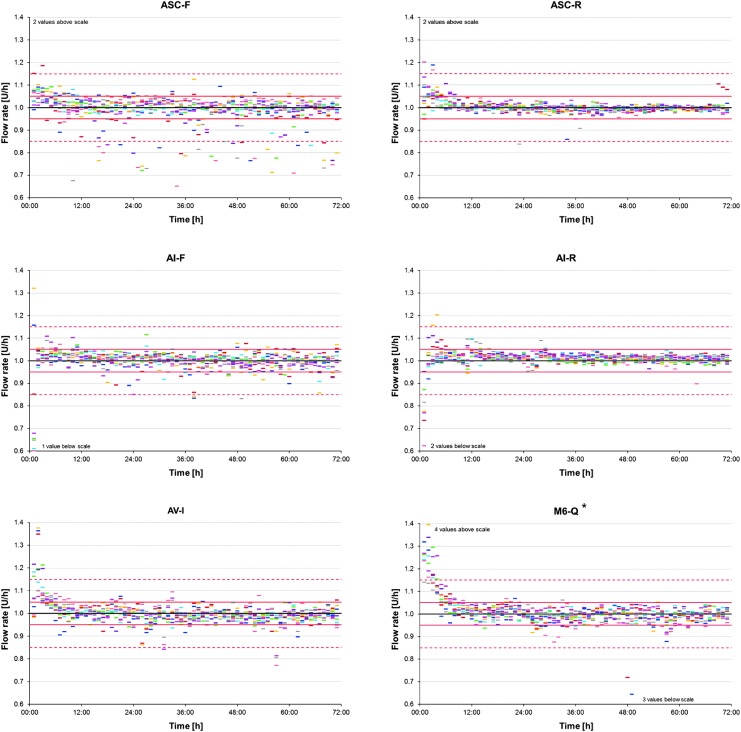

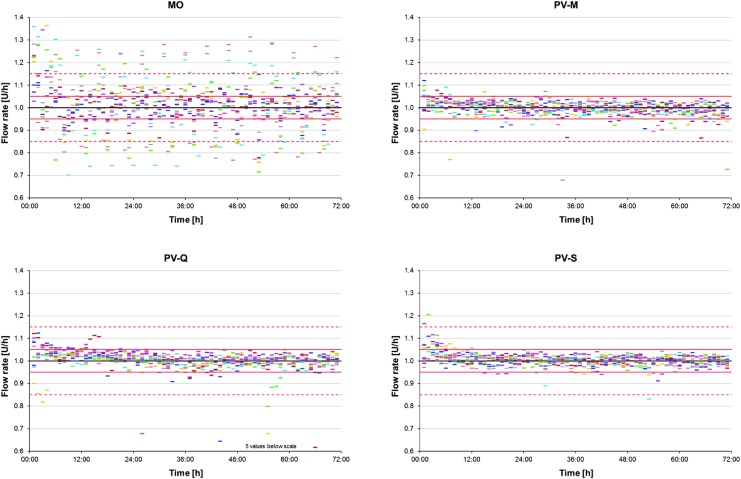
Basal rate accuracy of 1-h windows in the course of time for each insulin pump system. Colored dashes show the flow rate of 1-h windows calculated from weight increases for the individual runs (*n* = 71 values for each of 9 runs), the black line shows the target rate (1.0 U/h), and red lines and red dashed lines show the ±5% and ±15% range, respectively. *The batch used in this study was recalled by the manufacturer (Medtronic reference FA785) after the investigation.

**FIG. 4.**
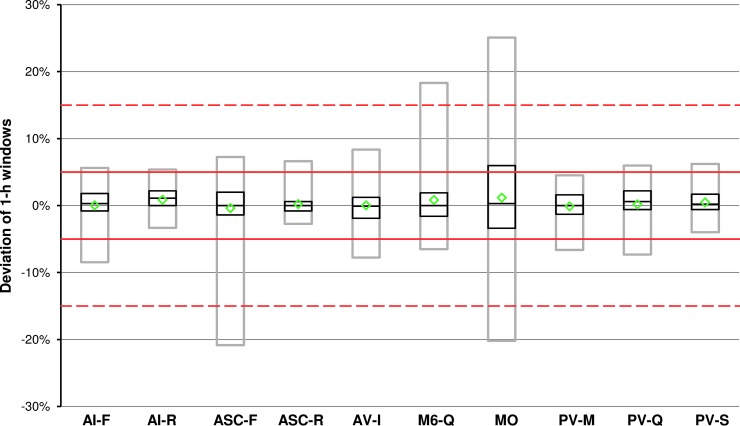
Basal rate accuracy of 1-h windows of all insulin pump systems at 1.0 U/h. For each system, mean (green diamonds), median with first and third quartile (black boxes), and 95% range (gray boxes) are shown (*n* = 648 values). Red lines and red dashed lines indicate target ±5% and target ±15%, respectively.

Figure 3 shows the calculated flow rate for the individual hours in the course of time for each repetition. A certain run-in time with larger deviations was observed for most systems, but to different degrees. It was most obvious for AV-I and M6-Q indicating initial overdelivery; AI-F and AI-R, however, rather showed initial underdelivery. ASC-F showed a biased scattering toward negative deviations during the whole test period. The highest precision was shown by ASC-R, whereas MO showed periodically alternating larger deviations that seemed to repeat after ∼5 h or 5 U (Figs. 3 and 4).

In general, individual runs of each insulin pump system were comparable with the exception of MO, which showed the described large amplitude in four of the nine runs. Means of all 1-h windows were within ±2% in all systems, and for 7 of the 10 systems ≥95% of 1-h windows were within ±15% of the intended basal rate (Fig. 4 and Table 5).

**Table 5.** Basal Rate (1.0 U/h) Accuracy: Percentage of 1-h Windows Within Different Deviation Thresholds (*n* = 693 Values)

*Pump system*	*1-h windows within ±15% (%)*	*1-h windows within ±10% (%)*	*1-h windows within ±5% (%)*
AI-F	98.3	96.7	89.2
AI-R	98.4	97.8	94.5
ASC-F	93.7	91.5	80.6
ASC-R	99.4	98.6	94.8
AV-I	97.8	96.4	85.4
M6-Q	95.6	93.1	84.0
MO	81.2	71.2	46.6
PV-M	99.5	98.7	92.6
PV-Q	98.3	96.7	89.8
PV-S	99.5	98.7	93.9

Trumpet curves for all pump systems as required by IEC 60601-2-24 are shown in Supplementary Figure S1.

## Discussion

In this comparative in vitro evaluation of insulin pump accuracy, 10 insulin pump systems, including 6 different pump models, were tested. To our knowledge, this is the largest comparison of insulin pumps tested in parallel published so far, the used methods were described and discussed in detail before.^9^ Factors such as the so-called siphon effect,^11^ infusion set changes,^12^ or pressure variations, which might have influenced the results or alleviated the inherent differences between the pumps, were avoided as far as possible. In practice, the tested insulin pumps might not be equally sensitive to these factors.

This study investigated accuracy at an intermediate basal rate and bolus doses as they might be used in adult CSII patients. Although the assumption that accuracy is higher the larger the delivered volume is could be confirmed in this study for two different bolus doses, the study results might not be transferable to all other bolus sizes or basal rates. There are no mandatory accuracy requirements or acceptance criteria for insulin pumps; however, under the assumption that a mean total deviation from target of ±5% is acceptable, all systems showed a good performance. At least for a 1.0 U/h basal rate, most manufacturers of the systems also describe an accuracy of ±5% in the respective manuals. Also, delivery time of a 10 U standard bolus in all pump systems complied with the delivery speeds given in the respective manuals. It has to be considered, however, that the described times differ between the particular insulin pump models, that is, that “standard” refers to a pump-specific feature that might be very fast in some and rather slow in other pump models. For pumps with adjustable bolus speed, for example, “standard” was the fastest of the options in AI and AV, whereas it was the slowest option in M6.

Variations between the individual pumps can only be seen when considering additional data beyond mean values. For bolus accuracy, this could be the evaluation of each single bolus instead of only the mean of several boluses, because this reflects the usage of boluses in clinical practice much better. In addition, variability between individual devices of the same model and IIS should be considered. Precision of single boluses was higher for the 10 U bolus, but also with 1 U, all boluses of most systems were within ±15% of the intended volume. One 1 U bolus each of AV-I and PV-Q, and several boluses of MO were outside these limits, but since these were evenly distributed in terms of negative and positive deviations, this could not be seen in the mean deviation. Using other methods, a high bolus delivery accuracy for MO was reported.^4^ Large differences between single boluses could be an issue in clinical practice for patients, as at one time an overdelivery and at the next time an underdelivery may lead to very different glycemic outcomes after the given bolus without being comprehensible for the patient.

Regarding basal rate accuracy, the total mean deviation is helpful to identify a possible general bias, but details such as whether delivery is constant over time or accuracy within shorter periods are missing. The first aspect was taken into account by dividing the total measurement period into different phases and comparing the mean deviation of these. Most obvious was the course of deviations during the first 12 h of the experiment. Although all systems were correctly installed and primed, for most of the tested systems, a clear run-in phase with less accurate delivery was observed. Commonly, this run-in phase was characterized by an over- or underdelivery that decreased with increasing run time, but duration and extent of the run-in were not specifically quantified in this study. Some pump systems, in particular ASC-R, AV-I, M6-Q, MO, and PV-S, showed a clear tendency toward an initial overdelivery, whereas AI-R showed initial underdelivery and for AI-F, ASC-F, PV-M, and PV-Q no clear conclusion could be drawn. Accordingly, in the first 12 h absolute deviations from the expected rate were larger than in the following 60 h. In a clinical setting, the first 12 h would be as important for the patient as the following 60 h and patients often anecdotally report changes in their glycemic control immediately after a change of the insulin pump system. One might argue that the test setting affected the run-in phase, but since the different pumps behaved differently within the same setting, it is likely caused by the specific insulin pump. Jahn et al. showed an initial underdelivery for all pumps tested in their study.^5^ For bolus delivery, no pattern of less accurate delivery during the first boluses of a series was observed.

Deviation of individual 1-h windows gives information about the continuity and accuracy for shorter time. A certain “oscillation” may be assumed among all pumps, as the measurement frequency might not have fit the interval of single basal rate bursts given by all pumps. However, most systems showed only a few 1-h windows outside a ±15% range, as already reported by Borot et al.^6^ The pronounced differences between individual 1-h windows of the patch pump MO are most likely due to pump-specific characteristics. Similar results, that is, large deviations between single doses of this pump were already observed by others.^5,6^

Trumpet curves as required by IEC 60601-2-24 were also provided; however, these curves might be misleading as they are often misunderstood.^13^ In addition, they exclude the first 24 h of delivery and only provide minimal and maximal deviations that might not be representative for the majority of values.

Knowledge about the impact of insulin delivery accuracy is limited; to our knowledge, there are no clinical studies that specifically investigated the effects of dosing errors on clinical outcomes. However, there are simulation studies for glucose measurement errors^14,15^ and clinical studies for carbohydrate counting errors^16,17^ that might lead to the assumption that dosing errors of >15% to 20% might have a clinical impact. A recent simulation study^18^ about the minimum insulin dose required for clinically meaningful changes in glucose concentrations suggests that this clinical impact may already be visible with basal rate changes of 0.1 U/h or bolus doses of 0.3 U for a time span of 4 h. With this study, the magnitude of insulin dosing errors was quantified; and similar model calculations based on these results, and possibly also for shorter time spans, could be helpful to quantify the clinical effects.

Whether the differences between the pumps and infusion sets observed in this study will also be noticed by patients when using the pumps will have to be addressed in further investigations. Likewise, the impact of different environmental factors that arise during use in practice on delivery accuracy should be studied.

This study has some limitations. First, comparability between the patch pump and the durable pumps is still limited due to different test setups, although an adequate setup for patch pumps was established in a prestudy.^9^ In addition, not for all durable pumps entirely comparable IIS were available. However, the obtained data do not indicate a profound influence of the particular IIS.

Basal rate accuracy results for M6-Q have to be regarded with caution, because after the tests were finished, the used batch of the infusion set was recalled by the manufacturer. The reason was a possible overdelivery in case liquid reaches the connection between IIS and cartridge during the priming process. It cannot be excluded that the initial deviations observed for M6-Q were caused by such a failure; however, measurements were not repeated since the recall came after the test and, therefore, at the time of the test, the used system reflected one that could also have been used by a patient.

Results of this study comply with those of other comparative studies in which, even if performed with variations in methodology, tested durable pumps showed a higher level of accuracy, especially when regarding short-term basal rate accuracy, than the patch pump tested in this study.^5,6^ However, in this study rather large doses were evaluated and with smaller doses, accuracy is expected to be lower.

## Conclusion

This technical evaluation of insulin pump accuracy regarding insulin delivery at medium bolus doses and basal rate showed that, in general, all compared systems showed a similar level of accuracy. Differences, especially between durable pumps and the patch pump, were observed when considering each hour of delivery separately.

## Supplementary Material

Supplemental data
