# Supplementary material for: Accuracy of Bolus and Basal Rate Delivery of Different Insulin Pump Systems
Source: Diabetes Technol Ther. 2019 Mar 30;21(4):201–8. doi: 10.1089/dia.2018.0376 (PMC6477586; doi:10.1089/dia.2018.0376)
Supplement: Supplemental data [file Supp_Fig1.pdf]

# Supplementary Data

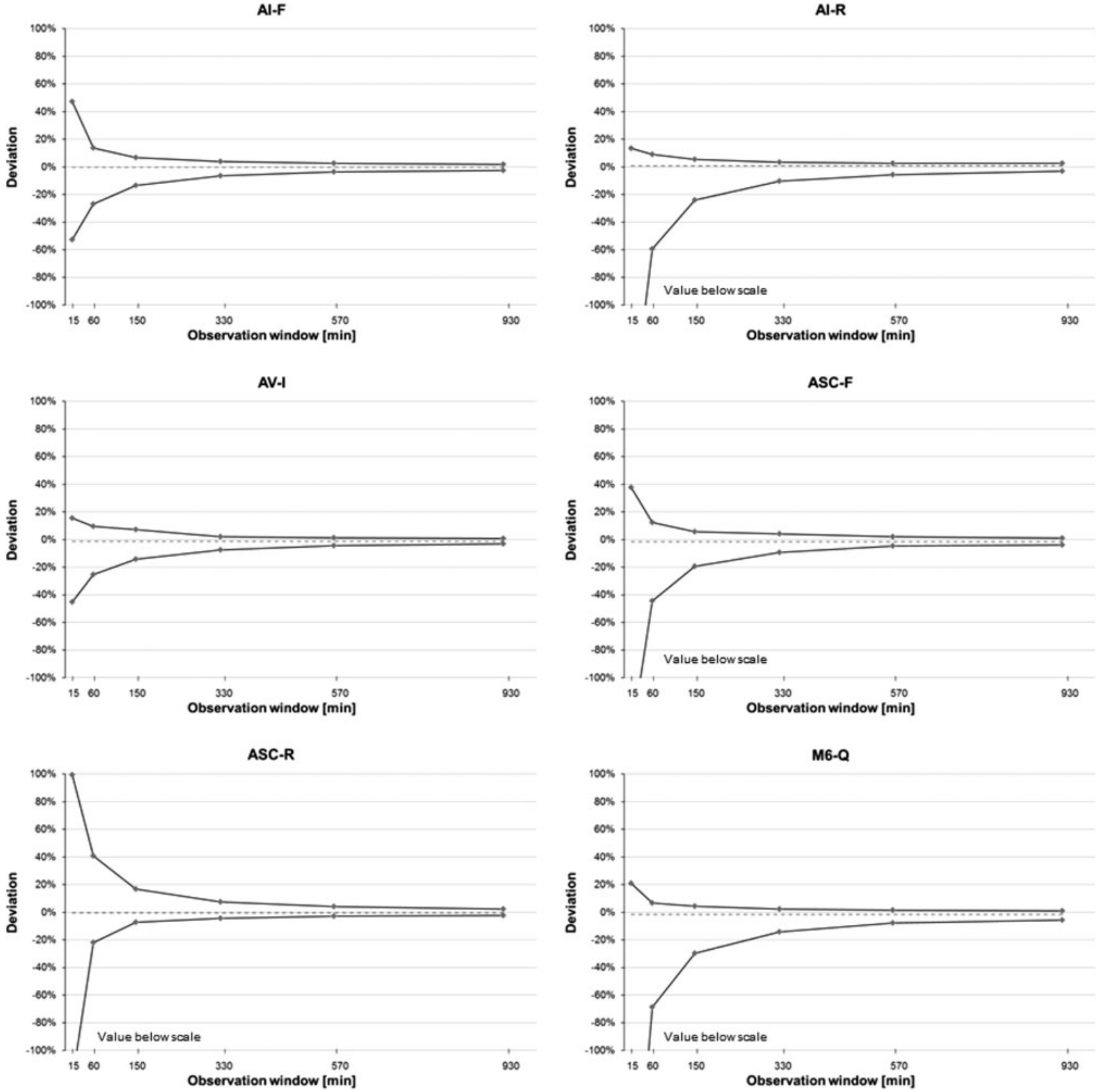


**SUPPLEMENTARY FIG. S1.** (Continued next page).

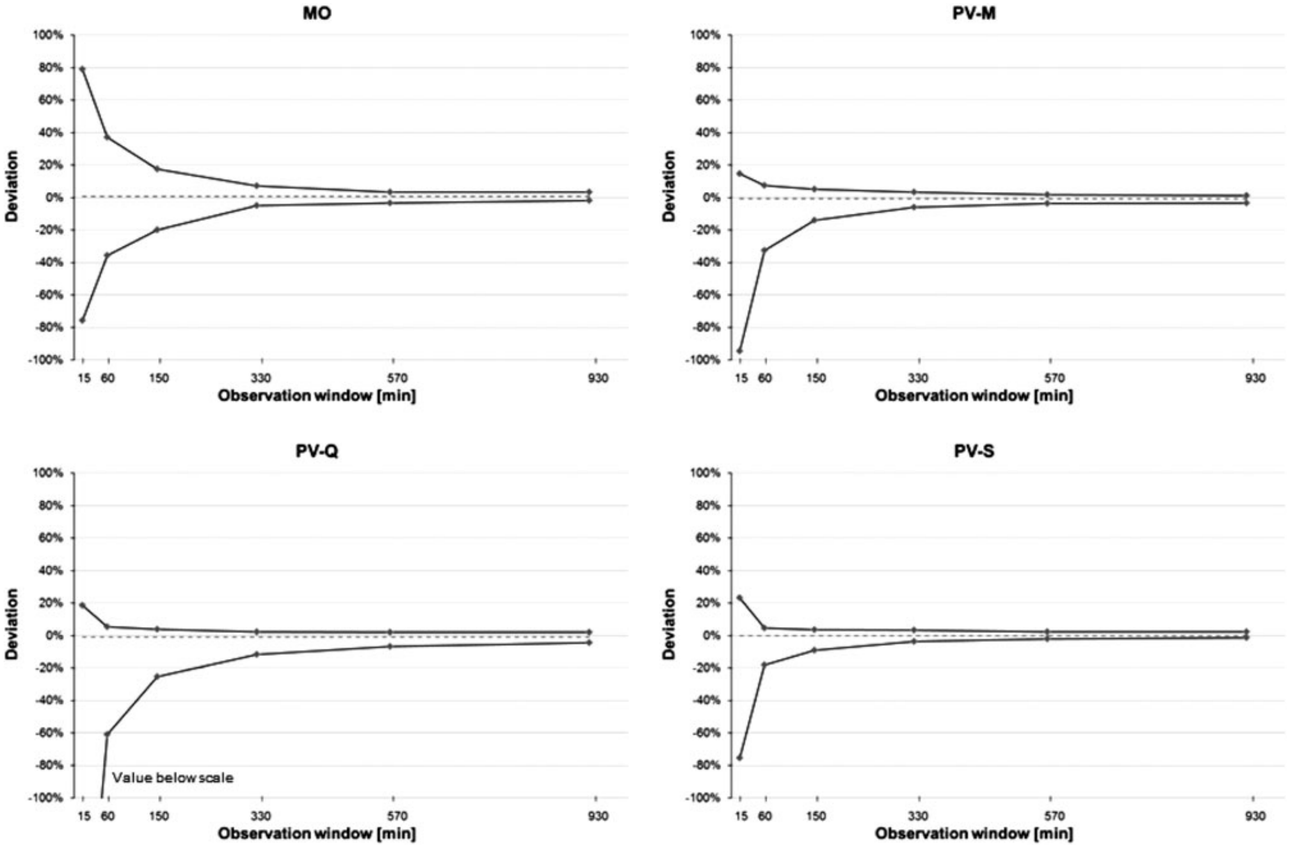


**SUPPLEMENTARY FIG. S1.** Trumpet curves according to IEC 60601-2-24 (first 24 h excluded) for a basal rate of 1 U/h. Connected dots show the minimal and maximal deviation of the nine data sets per insulin pump system within each observation window. Dotted lines show the total deviation for hours 25–72.
